# Muscle strength and activity in men and women performing maximal effort biceps curl exercise on a new machine that automates eccentric overload and drop setting

**DOI:** 10.1007/s00421-023-05157-9

**Published:** 2023-03-01

**Authors:** James L. Nuzzo, Matheus D. Pinto, Kazunori Nosaka

**Affiliations:** Centre for Human Performance, School of Medical and Health Sciences, Edith Cowan University, 270 Joondalup Drive, Joondalup, WA 6027 Australia

**Keywords:** Bicep curl, Eccentric, Muscle fatigue, Resistance exercise, Sex difference

## Abstract

**Purpose:**

Connected adaptive resistance exercise (CARE) machines are new equipment purported to adjust resistances within and between repetitions to make eccentric (ECC) overload and drop sets more feasible. Here, we examined muscle strength, endurance, electromyographic activity (EMG), and perceptions of fatigue during unilateral bicep curl exercise with a CARE machine and dumbbells. We also tested for sex differences in muscle fatigability.

**Methods:**

Twelve men and nine women attempted 25 consecutive coupled maximal ECC–concentric (CON) repetitions (ECC_max_–CON_max_) on a CARE machine. Participants also completed a CON one repetition maximum (1RM) and repetitions-to-failure tests with 60 and 80% 1RM dumbbells.

**Results:**

Maximal strength on the CARE machine was greater during the ECC than CON phase, illustrating ECC overload (men: 27.1 ± 6.8, 14.7 ± 2.0 kg; women: 16.7 ± 4.7, 7.6 ± 1.4 kg). These maximal resistances demanded large neural drive. Biceps brachii EMG amplitude relative to CON dumbbell 1RM EMG was 140.1 ± 40.2% (ECC) and 96.7 ± 25.0% (CON) for men and 165.1 ± 61.1% (ECC) and 89.4 ± 20.4% (CON) for women. The machine’s drop setting algorithm permitted 25 consecutive maximal effort repetitions without stopping. By comparison, participants completed fewer repetitions-to-failure with the submaximal dumbbells (e.g., 60%1RM—men: 12.3 ± 4.4; women: 15.6 ± 4.7 repetitions). By the 25th CARE repetition, participants reported heightened biceps fatigue (~ 8 of 10) and exhibited large decreases in ECC strength (men: 63.5 ± 11.6%; women: 44.1 ± 8.0%), CON strength (men: 77.5 ± 6.5%; women: 62.5 ± 12.8%), ECC EMG (men: 38.6 ± 20.4%; women: 26.2 ± 18.3%), and CON EMG (men: 36.8 ± 20.4%; women: 23.1 ± 18.4%).

**Conclusion:**

ECC overload and drop sets occurred automatically and feasibly with CARE technology and caused greater strength and EMG loss in men than women.

**Supplementary Information:**

The online version contains supplementary material available at 10.1007/s00421-023-05157-9.

## Introduction

Strength and conditioning coaches believe eccentric (ECC) resistance exercise is important for preventing and rehabilitating injuries and increasing muscle strength among athletes (Drury et al. 2021; Harden et al. 2020; McNeill et al. 2020). Consequently, coaches prescribe ECC resistance exercise, including accentuated ECC resistance exercise (i.e., eccentric overload) (Drury et al. 2021; Harden et al. 2020; McNeill et al. 2020; Weldon et al. 2022). To deliver ECC resistance exercise to athletes, coaches often rely on free weights or plate-loaded machines (Drury et al. 2021; Harden et al. 2020; McNeill et al. 2020). With such equipment, the load remains generally constant within and between repetitions, whereas muscle strength is ~ 40% greater during than the ECC (active muscle lengthening) than concentric (CON, active muscle shortening) repetition phase (Nuzzo et al. 2022). This incongruity between muscle strength capacity and resistance exercise equipment causes practical inconveniences for coaches. For example, when delivering accentuated ECC resistance exercise with free weights or plate-loaded machines, coaches might need to use supplementary devices such as “releasers,” which are difficult to use beyond the first repetition (Harden et al. 2020; Wagle et al. 2017). Thus, resistance exercise equipment can be a barrier to the delivery of ECC resistance exercise (Drury et al. 2021; Harden et al. 2020; Weldon et al. 2022).

Some equipment has been developed for the specific purpose of delivering ECC overload (Tinwala et al. 2017). However, limitations often exist in terms of machine size and weight and the number of exercises that can be performed (Tinwala et al. 2017). Flywheel machines, which are used by 7–30% of coaches (Drury et al. 2021; Harden et al. 2020; McNeill et al. 2020), are probably the most practical and affordable of such machines. Nevertheless, ECC loading with flywheels depends on the athlete’s CON phase performance (Suchomel et al. 2019). Thus, ECC overload might not occur for some ECC contractions, or it might occur through only a limited portion of the ECC phase. Moreover, coaches are unable to control the amount and timing of ECC overload with flywheel machines. Isokinetic dynamometers can also be used to passively move a limb through the CON phase and permit ECC-only contractions. However, factors such as machine size and cost and the types of exercise that can be performed limit the use of traditional isokinetic machines among coaches (Drury et al. 2021; Harden et al. 2020; McNeill et al. 2020).

Connected adaptive resistance exercise (CARE) machines, sometimes called “digital weights” or “intelligent loads”, are emerging technologies that appear to overcome some of the aforementioned limitations of traditional resistance exercise equipment and, thus, might facilitate the delivery of ECC resistance exercise (Nuzzo and Nosaka 2022). CARE machines integrate software with hardware to provide a resistance that adjusts in real time and in response to a participant’s volitional force and velocity within and between exercise repetitions. Nuzzo and Nosaka (2022) reported preliminary results on use of a CARE machine in one male participant. During coupled maximal ECC, maximal CON contractions (ECC_max_–CON_max_) of the biceps curl exercise, the machine provided an accentuated ECC resistance and automatically reduced the resistance to accommodate muscle fatigue (i.e., drop setting) (Nuzzo and Nosaka 2022). Such automatic drop setting has potential to overcome another limitation of most resistance exercise equipment, which is that the exercise set necessarily terminates at the point of CON phase failure. The only way an exercise set can continue is if a “spotter” helps the athlete lift the load in the CON phase (i.e., “forced repetitions”) (Hackett et al. 2013) or if the athlete stops and reduces the load by removing weight plates. Nuzzo and Nosaka's (2022) preliminary results suggest that CARE technology has potential to enhance the feasibility of both accentuated ECC and drop set resistance exercise for coaches. However, studies with larger samples are required.

Therefore, the purpose of the current study was to describe muscle strength, endurance, electromyographic activity (EMG), and perceptions of fatigue during unilateral bicep curl exercise on a CARE machine. The specific aims were to test, in a sample larger than previously studied (Nuzzo and Nosaka 2022), whether the machine effectively automates the delivery of ECC overload and drop setting, and whether maximal contractions on the machine demand levels of neural drive to the muscle that are comparable to that observed during a one repetition maximum (1RM) test with a dumbbell. Moreover, because roughly equal numbers of men and women volunteered for the experiment, and calls exist for more sex-segregated data in exercise research (Schilaty et al. 2018), we explored potential sex differences in muscle fatigability. Women appear to be less susceptible to muscle fatigue than men during isometric tasks (Hunter 2014, 2016a). This sex difference has been attributed to factors such as muscle size, fiber type, metabolism, and contractile function (Hunter 2014, 2016a). However, whether sex differences in muscle fatigability exist during *non*-isometric is unclear due to less research on the topic (Hunter 2016b). For example, whether a difference exists in the number of repetitions that men and women can complete at equal relative loads remains uncertain due to mixed findings (Nuzzo 2023). Resistance exercise programs typically consist of coupled ECC–CON contractions rather than isometric contractions. Thus, additional research on men and women performing coupled ECC–CON contractions can reveal if sex differences exist in the magnitude of strength loss experienced during fatiguing maximal effort exercise; the number of repetitions that can be completed with submaximal loads; and the extent to which muscle fatigue and pain are perceived during exercise.

## Methods

### Overall design

Twenty-one participants performed unilateral bicep curl exercise with their right upper-limb in two familiarization sessions and one testing session. Sessions were separated by ~ 7 days and occurred at roughly the same time of day. In the first familiarization session, participants completed a bicep curl 1RM with a dumbbell and practiced bicep curl exercise on the CARE machine. In the second familiarization session, participants repeated the dumbbell 1RM test, and practiced repetitions-to-failure tests with 60 and 80% 1RM dumbbell loads and completed one set of exercise on the CARE machine. After the two familiarization sessions, in one testing session, participants completed the following: dumbbell 1RM test, repetitions-to-failure tests with 60 and 80% 1RM dumbbell loads in random order, and one set of 25 ECC_max_–CON_max_ repetitions on the CARE machine. Study outcomes included muscle strength, repetitions completed, root mean square (RMS) amplitude of the EMG signal, and perceptions of fatigue and pain. For reasons stated earlier, we also explored potential sex differences in these outcomes.

### Participants

Twelve men and nine women participated in the study (Table 1). They were similar in age (30.2 ± 7.7 vs 30.6 ± 8.4 y; mean ± SD), current resistance exercise frequency (2.9 ± 1.8 vs 3.7 ± 1.3 days/wk), and years of resistance exercise experience (8.4 ± 7.5 vs 9.9 ± 8.9 y). The men had taller body heights (180.6 ± 7.7 vs 167.8 ± 5.4 cm), greater body masses (83.8 ± 14.7 vs 62.1 ± 6.9 kg), and less range of motion about the right elbow (131.2 ± 10.1 vs 142.3 ± 8.7°). Participants were recruited through word-of-mouth and advertisements posted on social media and at a local fitness center. Participants were required to have no previous injury to their right upper-limb and no contraindications to exercise as determined by the Physical Activity Readiness Questionnaire (2020 PAR-Q +) (Warburton et al. 2011). Most individuals were participating in resistance exercise or had participated in resistance exercise in the past. Sample sizes were comparable to similar investigations (Baudry et al. 2007; Pasquet et al. 2000). The study protocol (2021-02621-NUZZO) was approved by the Human Research Ethics Committee at Edith Cowan University. All individuals provided written informed consent and were remunerated for participation. All sessions were conducted by a certified strength and conditioning specialist.

**Table 1:** Results summary of muscle strength, endurance, and activity, and perceptions of fatigue in men and women during unilateral bicep curl exercise with a connected adaptive resistance exercise machine and dumbbell loads

Outcome	Men (*n* = 12)	Women (*n* = 9)	Effect size of sex difference
	Mean ± SD	Mean ± SD	Hedges *g* [95% CI]
Maximal muscle strength			
DB bicep curl 1RM (kg)	22.6 ± 5.7	12.5 ± 1.7	2.169 [1.076, 3.227]
DB bicep curl 1RM-to-body mass ratio	0.27 ± 0.05	0.20 ± 0.04	1.384 [0.432, 2.308]
CARE peak ECC strength (kg)	31.2 ± 7.1	18.8 ± 1.7	2.142 [1.055, 3.196]
CARE peak CON strength (kg)	25.3 ± 7.1	14.6 ± 3.1	1.788 [0.769, 2.776]
CARE peak ECC:CON strength ratio	1.26 ± 0.24	1.35 ± 0.33	− 0.298 [− 1.130, 0.540]
CARE highest average ECC strength (kg)	27.1 ± 6.8	14.7 ± 2.0	2.228 [1.123, 3.298]
CARE highest average CON strength (kg)	16.7 ± 4.7	7.6 ± 1.4	2.348 [1.218, 3.444]
CARE highest average ECC:CON strength ratio	1.66 ± 0.31	1.98 ± 0.41	− 0.875 [− 1.739, 0.010]
Maximal muscle activity (EMG) on CARE machine			
ECC biceps brachii RMS amp (% CON DB 1RM)	140.1 ± 40.2	165.1 ± 61.1	− 0.478 [− 1.315, 0.371]
CON biceps brachii RMS amp (% CON DB 1RM)	96.7 ± 25.0	89.4 ± 20.4	0.301 [− 0.538, 1.133]
ECC brachioradialis RMS amp (% CON DB 1RM)	129.7 ± 56.7	146.0 ± 43.0	− 0.305 [− 1.137, 0.534]
CON brachioradialis RMS amp (% CON DB 1RM)	91.3 ± 27.2	98.3 ± 28.9	− 0.240 [− 1.070, 0.596]
Muscle fatigability			
Muscle endurance performance			
Repetitions-to-failure with 60% DB 1RM	12.3 ± 4.4	15.6 ± 4.7	− 0.684 [− 1.533, 0.182]
Repetitions-to-failure with 80% DB 1RM	6.5 ± 2.7	6.2 ± 2.1	0.108 [− 0.724, 0.937]
CARE ECC_max_–CON_max_ repetitions completed	25. 0 ± 0	24.9 ± 0.3	0.493 [− 0.357, 1.331]
Strength loss from CARE			
ECC strength (% decrease)	63.5 ± 11.6	44.1 ± 8.0	1.827 [0.801, 2.822]
CON strength (% decrease)	77.5 ± 6.5	62.5 ± 12.8	1.483 [0.515, 2.421]
EMG loss from CARE			
ECC biceps brachii RMS amp (% decrease)	38.6 ± 25.8	26.2 ± 18.3	0.521 [− 0.331, 1.360]
CON biceps brachii RMS amp (% decrease)	36.8 ± 20.4	23.1 ± 18.4	0.672 [− 0.193, 1.520]
ECC brachioradialis RMS amp (% decrease)	42.6 ± 16.7	30.4 ± 10.8	0.803 [− 0.074, 1.661]
CON brachioradialis RMS amp (% decrease)	38.9 ± 17.0	24.4 ± 16.8	0.824 [− 0.056, 1.683]
Perceived biceps fatigue (0–10 scale)			
Baseline	0.1 ± 0.3	0.2 ± 0.4	− 0.370 [− 1.230, 0.473]
After DB 1RM	2.4 ± 2.3	2.7 ± 1.4	− 0.109 [− 0.938, 0.722]
After 80% DB 1RM repetitions-to-failure	4.9 ± 1.7	4.7 ± 1.7	0.126 [− 0.706, 0.955]
After 60% DB 1RM repetitions-to-failure	5.3 ± 1.7	6.9 ± 1.4	− 0.965 [− 1.838, − 0.070]
After 25 CARE ECC_max_–CON_max_ repetitions	7.8 ± 1.9	8.2 ± 1.4	− 0.214 [− 1.043, 0.622]
Perceived arm strength capacity (0–100% scale)			
Baseline	95.4 ± 4.7	90.3 ± 7.3	0.819 [− 0.060, 1.678]
After DB 1RM	73.8 ± 20.2	68.6 ± 19.2	0.256 [− 0.581, 1.086]
After 80% DB 1RM repetitions-to-failure	48.2 ± 24.1	43.3 ± 15	0.223 [− 0.613, 1.053]
After 60% DB 1RM repetitions-to-failure	44.8 ± 23.4	35.0 ± 17.7	0.446 [− 0.401, 1.282]
After 25 CARE ECC_max_–CON_max_ repetitions	7.8 ± 12.2	3.4 ± 4.0	0.393 [− 0.451, 1.227]
Perceived biceps pain (0–10 scale)			
Baseline	0.0 ± 0.0	0.1 ± 0.3	− 0.493 [− 1.331, 0.357]
After DB 1RM	0.8 ± 1.3	1.1 ± 1.1	− 0.218 [− 1.047, 0.618]
After 80% DB 1RM repetitions-to-failure	2.1 ± 2.1	3.2 ± 2.3	− 0.501 [− 1.339, 0.350]
After 60% DB 1RM repetitions-to-failure	2.5 ± 2.5	4.1 ± 2.5	− 0.612 [− 1.456, 0.247]
After 25 CARE ECC_max_–CON_max_ repetitions	4.0 ± 3.8	5.9 ± 3.1	− 0.512 [− 1.351, 0.340]

*1RM* one repetition maximum, *CARE* connective adaptive resistance exercise machine, *CON* concentric, *DB* dumbbell, *ECC* eccentric, *RMS* root mean square amplitude

### Procedures

#### Electromyography and electrogoniometry

Prior to exercise, the right upper-limb was prepared for EMG. The skin over the biceps brachii, brachioradialis, and anterior deltoid was shaved, abraded, and cleansed with alcohol. Wireless surface electrodes were placed on the skin (Trigno Wireless System, Delsys, Natick, USA; sampling rate: 2148 Hz; bandwidth frequency: 20–450 Hz). The biceps brachii electrode was placed along the longitudinal midline and over the distal-most portion of the muscle when the elbow angle was 90°. For brachioradialis and anterior deltoid, electrodes were placed over the most distal portion of the muscle bellies as determined by palpation. All electrodes were secured to the skin with tape. The EMG signals were acquired with electrode manufacturer’s software (Delsys EMGworks Analysis 4.7.9).

An electrogoniometer was used to measure elbow joint angle during exercise (Biometrics Ltd, Ladysmith, USA; sampling rate: 519 Hz; gain: 1400 dB). The goniometer consisted of two endblocks connected by a wire that had strain gauges mounted around it, with the wire protected by an outer spring. Using double-sided tape, the proximal and distal endblocks were secured to the medial sides of the humerus and forearm, respectively. The mid-point of the spring was positioned at the medial epicondyle of the humerus.

#### Maximum dumbbell curl

Dumbbell tests were completed with a short bar and weight plates, which could be incremented by 0.5 kg (Xpeed, Mawson Lakes, Australia). The investigator handed the dumbbell to the participant who stood upright with the forearm supinated. The 1RM tested CON strength, which was the maximal load the participant could curl one time. Previous knowledge of the participant’s 1RM was available to the investigator from two familiarization sessions. The first 1RM attempt was the best 1RM from familiarization. Loads were changed, either by 0.5 or 1.0 kg, until the 1RM was achieved, usually within three attempts. A 5-min rest was given between attempts. Participants were not permitted to perform excessive lumbar extension or shoulder flexion to lift the load. The warm-up before the 1RM test was one set of three repetitions with a 60% 1RM dumbbell.

#### Repetitions-to-failure with 60 and 80% 1RM dumbbells

After completing the 1RM, participants were given a 15-min rest. Then, in random order, they completed repetition-to-failure tests with dumbbells equal to 60 and 80% 1RM. The dumbbell repetition-to-failure tests were included in the study, primarily, but not exclusively, as a proof of concept of the inherently different nature of constant load dumbbells versus CARE technology resistance and the impact these different forms of resistance have on exercise set termination. Loads of 60 and 80% 1RM were chosen because they represent moderate and heavy loads, fall within loading guidelines for resistance exercise prescriptions (American College of Sports Medicine 2009), and have been used in previous studies on repetitions-to-failures tests in men and women (Nuzzo 2023).

Each participant lifted and lowered the dumbbell until CON phase failure. The target repetition duration was 5-s: 2-s CON phase, 1-s pause at end of the CON phase, 2-s ECC phase, and no pause prior to the next CON phase. This repetition timing was chosen because, in pilot testing, this was the approximate timing for the type of repetition performed on the CARE machine. For example, the 1-s pause at the top of the dumbbell curl was incorporated because the CARE machine requires participants to pause at the top of the curl to receive ECC overload. A metronome (60 beats per minute) provided an auditory “beep” for participants to follow, and the investigator verbally counted the pace. In some instances, fatigue caused repetition slowing and participants could not maintain pace. The metronome was used to provide a general guide for pacing rather than to serve as a strict criterion. If all other aspects of exercise technique were maintained, repetitions in which a participant’s pace misaligned with the metronome pace were still counted. A 15-min rest was provided between the two repetition-to-failure tests and also prior to the protocol on the CARE machine.

#### CARE machine

The CARE machine (Trainer^+^, Vitruvian, Perth, Australia) consists of motorized winches that apply forces to two independent ropes that exit the top of the machine. The winches are controlled by a mobile phone application and machine software. Using a handle attached to one  rope, participants exerted force against the rope as the winch retracted it. Once exercise commenced, the machine’s algorithm adjusted resistances between 0 and 100 kg per rope in real time at a rate of 50 Hz. The magnitude of resistance adjustment depended on the participant’s force-generating capacity, movement velocity, exercise mode, and initial resistance selected.

We assigned participants the task of completing 25 consecutive ECC_max_–CON_max_ unilateral bicep curl repetitions on the machine without stopping. We chose 25 repetitions because this was the maximal number of repetitions allowed for one set in the machine’s mobile phone application. Thus, it served to test the limits of the machine. Also, based on previous preliminary work, this number of repetitions on the machine appeared appropriate for studying muscle fatigue (Nuzzo and Nosaka 2022).

Participants stood on the machine’s platform and grasped the handle attached to the right rope. Body position on the platform was such that the rope was perpendicular to the platform when the participant’s right elbow was 90° with the forearm supinated. At the start of exercise, participants performed three warm-up repetitions with little to no resistance. These repetitions allowed the machine to identify the movement ranges of the CON and ECC phases. A supramaximal resistance known to be ~ 2 kg higher than the participant’s ECC maximum strength on the machine from familiarization sessions was selected as the target resistance. This target resistance was the maximal resistance the participant would ever experience during the set.

The CARE machine adjusts resistances in real time to accommodate the participant’s force-generating capacity, so the machine does not place upon the participant a resistance that the participant cannot resist. Use of a supramaximal ECC resistance ensured that all ECC and CON contractions were performed with maximal effort. The machine then permitted these maximal ECC and CON resistances to be experienced if three behavioral conditions were met: (a) at the end of the CON phase, the participant paused ~ 1 s to allow a maximal ECC resistance to load; (b) the participant resisted the ECC resistance with maximal effort throughout the ECC phase; and (c) at the end of the ECC phase, the participant attempted to perform the CON phase as “hard and fast as possible.” When this latter instruction was followed, the participant started the CON phase by pulling against the last resistance experienced during the ECC phase. As ECC strength is greater than CON strength, the machine’s algorithm recognizes that the participant is unable to overcome the resistance based on slow movement velocity at the start of the CON phase. The machine, then, decreases the resistance to allow the participant to displace the handle, but it does so to a degree that high effort is required throughout the CON phase.

The machine’s default ECC–CON exercise mode uses an adjustable-resistance algorithm which incorporates a velocity band that is specific to the CON and ECC phases. Slow movement velocities below the bottom threshold of the band in the CON phase inform the machine that the participant is struggling to overcome the resistance. Consequently, the algorithm decreases the resistance in real time to allow the participant to complete the CON phase. If the participant’s movement velocity in the CON phase is above the upper threshold of the velocity band, the algorithm increases the resistance in real time to make the CON phase more difficult. During the ECC phase, slow movement velocities below the bottom threshold cause the algorithm to increase the resistance, as this implies the participant has the strength to withstand the load, whereas fast movement velocities above the upper threshold imply the participant does not have the strength to control the resistance, causing the algorithm to decrease the resistance.

The investigator provided verbal encouragement and instruction throughout the exercise protocol on the machine. The participant’s smartphone was positioned on a stand in front of them. The phone provided visual feedback of the machine’s resistance in real time. The investigator encouraged the participant to get the resistance numbers on the screen as high as possible.

#### Perceptions

Three scales were used to assess perceptions during exercise (Supporting Information 1). One scale assessed biceps fatigue: “How much fatigue do you feel in your biceps right now?” Scale anchors were 0 and 10. A second scale assessed arm strength capacity: “How much of your maximal strength capacity do you have in your right arm right now?” Scale anchors were 0 and 100%. A third scale assessed biceps pain: “How much discomfort/pain do you feel in your biceps right now?” Scale anchors were 0 and 10. Participants were allowed to reflect upon the questions how they wanted. Answers were recorded at the beginning of the testing session (baseline) and then just prior to and immediately after each test. During the CARE machine protocol, participants were asked their arm’s strength capacity after Repetitions 5, 10, 15, 20, and 25. For perceptions assessed before and after the exercise sets, participants often flexed and extended their elbow before answering.

### Data processing

Data from the CARE machine, EMG unit, and electrogoniometer were imported into Spike software for analysis (Cambridge Electronics Design, Cambridge, UK). Data from the electrogoniometer and rope from the CARE machine were used to identify the ECC and CON phases. With the CARE machine, average resistances or loads over the range of motions of the ECC and CON phases were measured for all repetitions. In the current study, each muscle contraction was performed with maximal effort, and the CARE machine adjusted the resistance in real time to accommodate the participant’s volitional force. Thus, in the current study, the CARE machine’s resistances reflected the maximal muscle strength of the participant. We use phrases such as “average strength” and “average load” interchangeably, and they refer to the average of all resistances sampled (50 Hz) over the entire movement ranges of the ECC and CON phases of a given repetition. Peak strength was the highest resistance sampled within repetitions 1–5 on the CARE machine. Average strength was the repetition within repetitions 1–5 that had the highest average resistance. This average resistance was considered the “initial” strength and was used in the computation of strength loss described below. The repetition within repetitions 1–5 on the CARE machine that had the highest raw EMG RMS amplitude was considered the “initial” muscle activity and was used to compare to muscle activity during the 1RM dumbbell test. “Final” muscle strength and EMG represent the average across repetitions 21–25. For descriptive purposes, raw EMG during use of the CARE machine was also expressed relative to raw EMG during the dumbbell 1RM test. Strength loss—the percent change in resistance from “initial” to “final” strength—was used as an index of muscle fatigue. Finally, the correlation (Pearson’s *r*) between participants’ dumbbell 1RM and initial CON phase average strength on the CARE machine was 0.964. This illustrates concurrent validity of the CARE machine resistances.

### Statistical analyses

We present individual participant data and supplement it with group means, standard deviations (SD), mean differences, effect sizes, and 95% confidence intervals (CI) (Cohen 1994; Dankel et al. 2017; Dankel 2020; McShane et al. 2019; Szucs and Ioannidis 2017; Weissgerber et al. 2015; Zhu 2012). Some data violated assumptions of normality and homogeneity of variances (Chen and Zhu 2001). Hedges *g* effect size [95% CI] was used to correct for bias in small independent samples and unequal variances when comparing results between men and women (Lakens 2013; Marfo and Okyere 2019). In the figures and table, 95% CIs of mean differences that do not include zero can be considered statistically significant (i.e., *p* < 0.05) (Cumming 2009). Version 28 of the Statistical Package for the Social Sciences (SPSS, Armonk, US) was used to analyze the data.

## Results

**Fig. 1:**
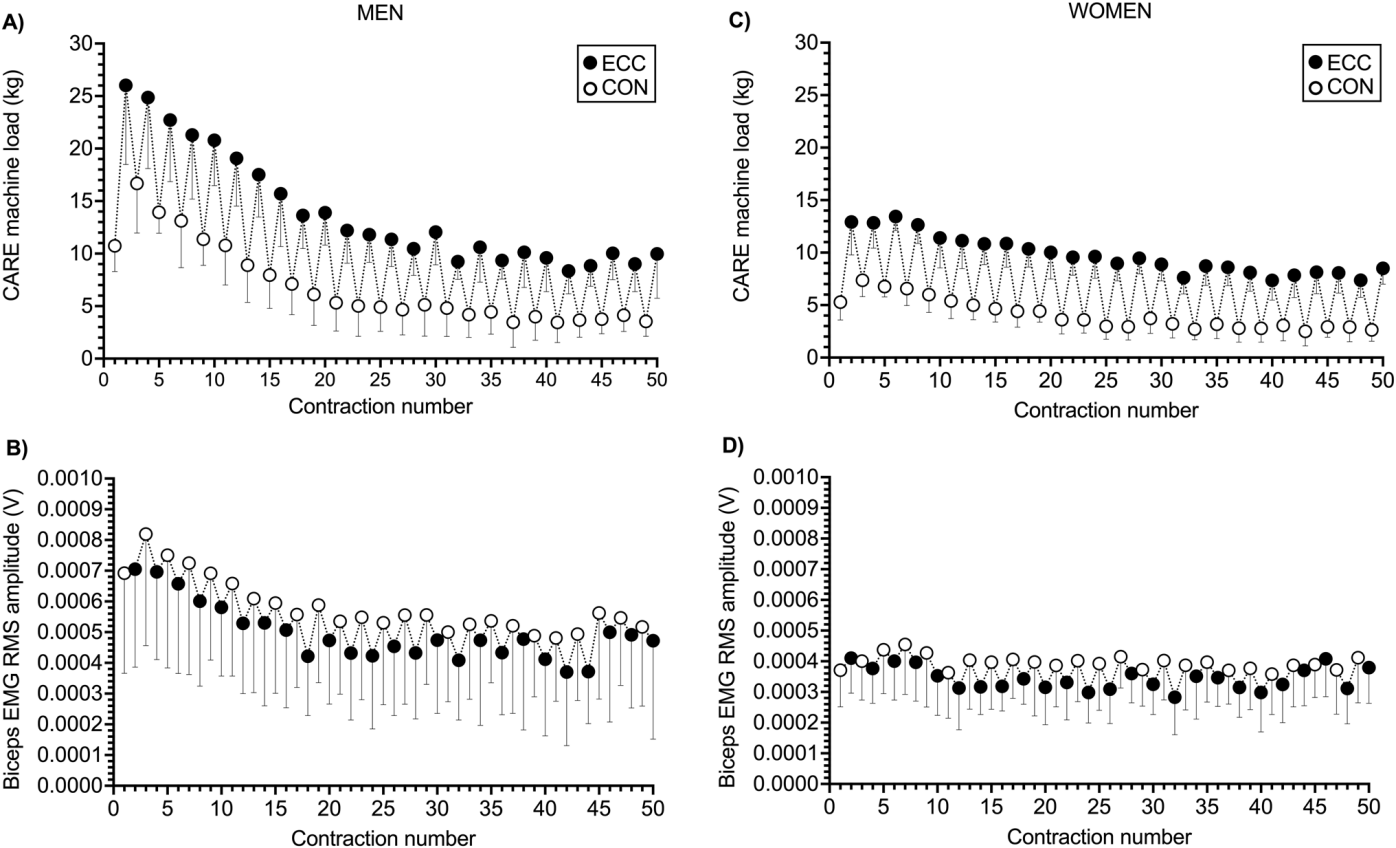
Eccentric (ECC, black circles) and concentric (CON, white circles) phase loads (kg) (**A**, **C**) and biceps brachii electromyographic (EMG) activity (**B**, **D**) during one set of 25 maximal eccentric, maximal concentric (ECC_max_–CON_max_) repetitions (50 contractions) of the unilateral bicep curl on the connected adaptive resistance exercise (CARE) machine. **A** For men, ECC phase strength was greater than CON phase strength. ECC and CON phase strength were reduced most markedly within the first 15 repetitions after which they tended to plateau. **B** For men, root mean square (RMS) amplitude of biceps brachii EMG was similar in the ECC and CON phases throughout exercise. Biceps brachii EMG amplitudes followed a trend similar to strength, with reductions most marked within the first 15 repetitions followed by a plateauing behavior. **C** For women, ECC phase strength were greater than CON phase strength. Both ECC and CON phase strength decreased progressively over the first 12 repetitions after which they tended to plateau. **D** For women, RMS amplitude of biceps brachii EMG was similar in the ECC and CON phases throughout exercise. Brachioradialis EMG amplitudes (not displayed) followed a similar trend to biceps brachii in both men and women. All data are presented as mean ± SD

**Fig. 2:**
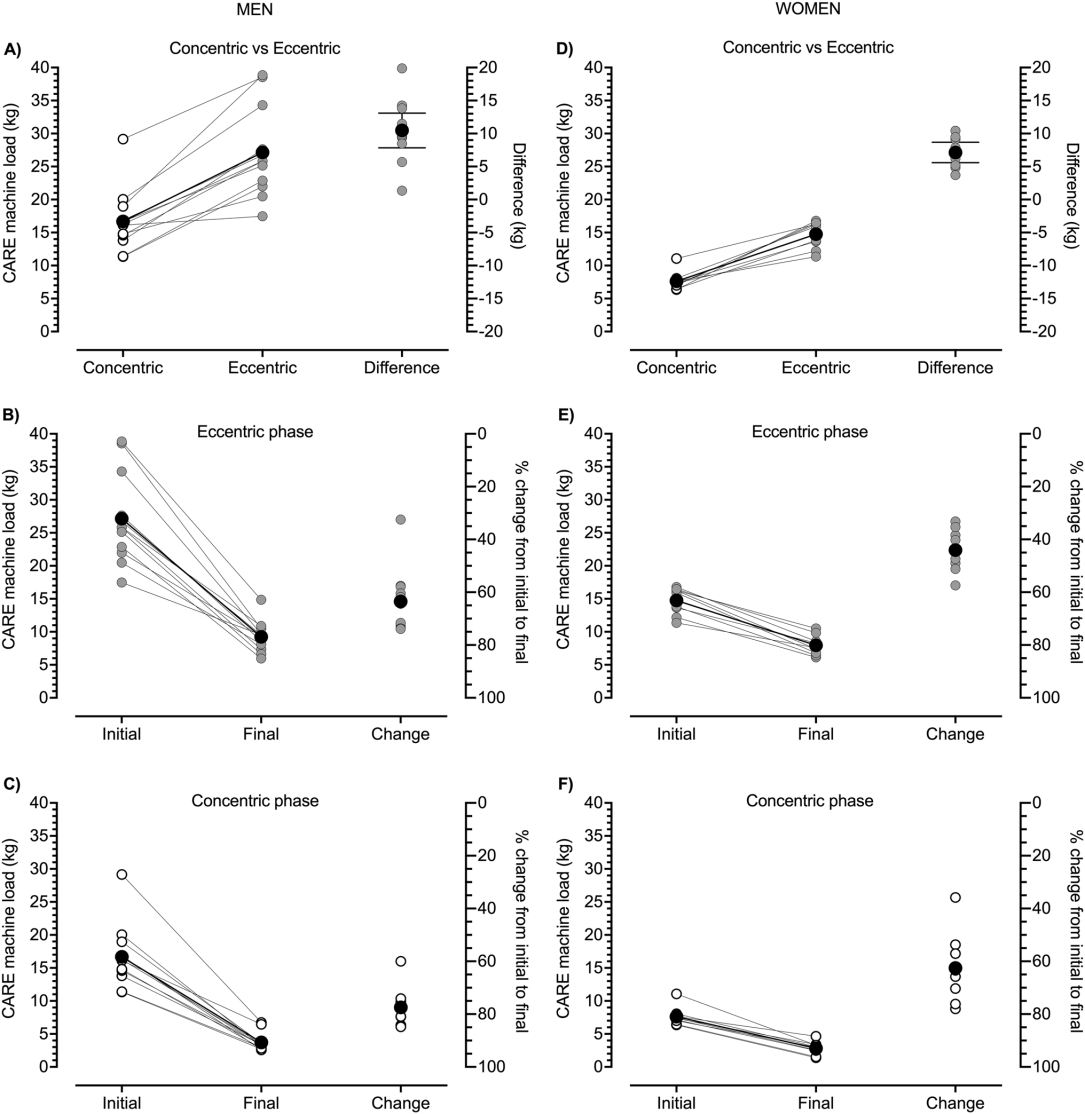
Group means (black circles) and individual participant data (white or gray circles) for highest average eccentric (ECC) (**A**, **B**, **D**, **E**) and concentric (CON) (**A**, **C**, **D**, **F**) phase loads at the start (initial) and end (final) of one set of 25 ECC_max_–CON_max_ repetitions of the unilateral bicep curl on the connected adaptive resistance exercise (CARE) machine. **A** Men exhibited greater average ECC than CON phase strength. The mean difference [95% CI] between ECC and CON phase strength at the start of exercise was 10.5 kg [7.6, 13.4 kg] (Hedges *g* [95% CIs] = 1.528 [0.696, 2.359]). **B** All men exhibited large reductions in ECC phase strength from the start to end of exercise. The mean percent loss in ECC phase strength was 64%. Not displayed is the mean raw difference between initial and final ECC phase strength which was − 17.9 kg [− 21.5, − 14.2 kg]. **C** All men exhibited large reductions in CON phase strength from the start to end of exercise. The mean percent loss in CON phase strength was 78%. Not displayed is the mean raw difference between initial and final CON phase strength which was − 12.9 kg [− 15.5, − 10.4 kg]. **D** Women exhibited greater average ECC than CON phase strength. The mean difference between ECC and CON phase strength at the start of exercise was 7.1 kg [5.4, 8.9 kg] (Hedges *g* = 3.669 [1.366, 5.972]). **E** All women exhibited large reductions in ECC phase strength from the start to end of exercise. The mean percent loss in ECC phase strength was 44%. Not displayed is the mean raw difference between initial and final ECC phase strength which was − 6.8 kg [− 8.2, − 5.4 kg]. **F** All women exhibited large reductions in CON phase strength from the start to end of exercise. The mean percent loss in CON phase strength was 63%. Not displayed is the mean raw difference between initial and final CON phase strength which was − 4.8 kg [− 5.8, − 3.8 kg]. See Table 1 for between-sexes effect sizes

**Fig. 3:**
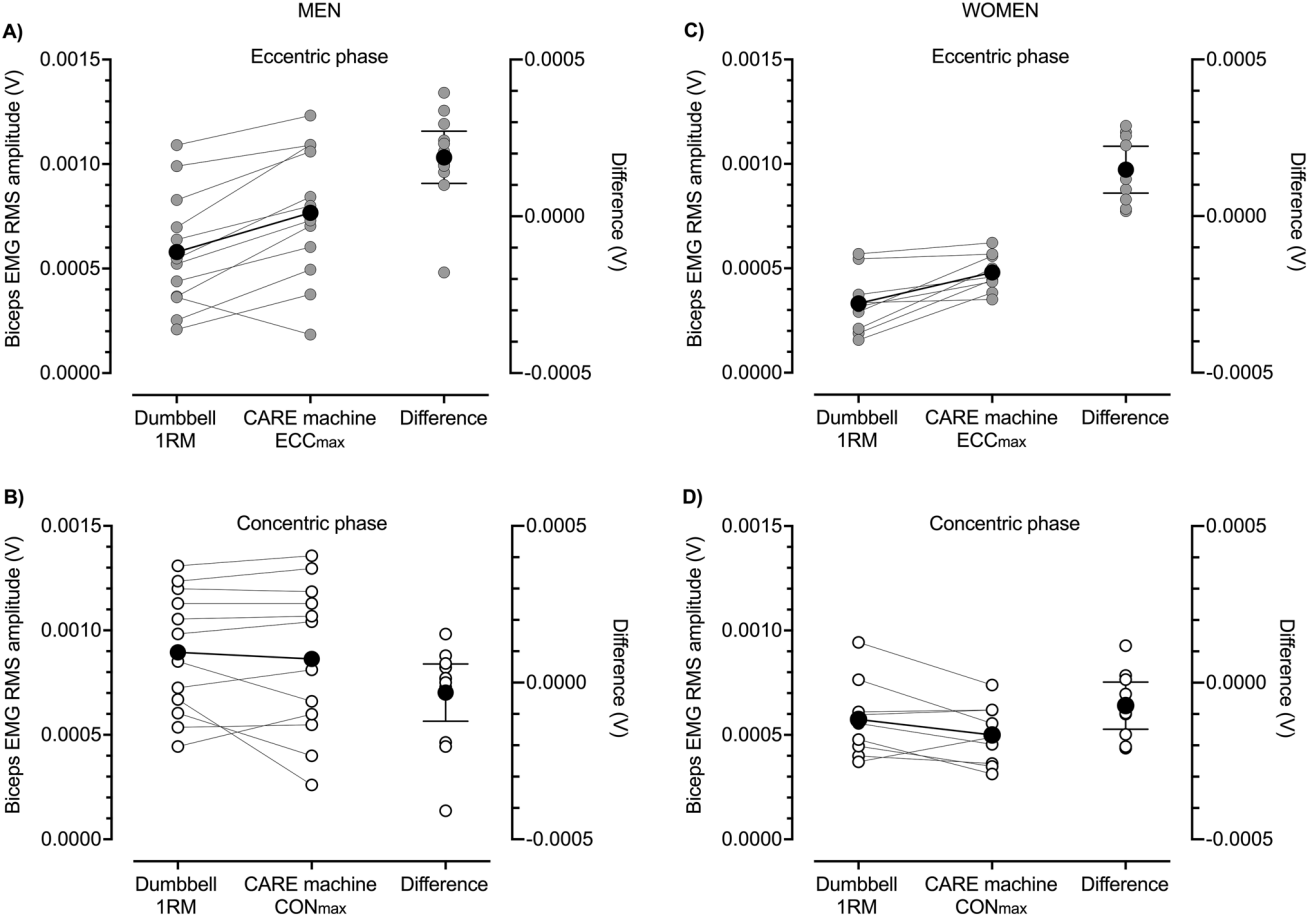
Means (black circles) and individual participant data (gray circles) for root mean square (RMS) amplitudes of biceps brachii electromyographic (EMG) activity during the eccentric (ECC) (**A**, **C**) and concentric (CON) (**B**, **D**) phases of the CON bicep curl one repetition maximum (1RM) with a dumbbell and during ECC_max_–CON_max_ repetitions on the connected adaptive resistance exercise (CARE) machine. **A** In men, biceps brachii EMG RMS amplitude during the ECC phase of ECC_max_–CON_max_ repetitions on the CARE machine was greater than during the ECC phase of the CON dumbbell 1RM (mean raw difference [95% CI] = 0.000188 V [0.000097, 0.000279 V]; Hedges *g* [95% CI] = 0.565 [0.184, 0.946]). **B** In men, biceps brachii EMG RMS amplitude during the CON phase of ECC_max_–CON_max_ repetitions on the CARE machine was similar to during the CON phase of the CON dumbbell 1RM (mean raw difference = − 0.000032 V [− 0.000505, − 0.000147 V]; Hedges *g* = − 0.081 [− 0.339, 0.176]). **C** In women, biceps brachii EMG RMS amplitude during the ECC phase of ECC_max_–CON_max_ repetitions on the CARE machine was greater than during the ECC phase of the CON dumbbell 1RM (mean raw difference = 0.000148 V [0.000062, 0.000233 V]; Hedges *g* = 1.005 [0.186, 1.825]). **D** In women, biceps brachii EMG RMS amplitude during the CON phase of ECC_max_–CON_max_ repetitions on the CARE machine was greater than during the CON phase of the CON dumbbell 1RM (mean raw difference = − 0.000073 V [− 0.000159, 0.000012 V]; Hedges *g* = − 0.379 [− 0.872, 0.115])

**Fig. 4:**
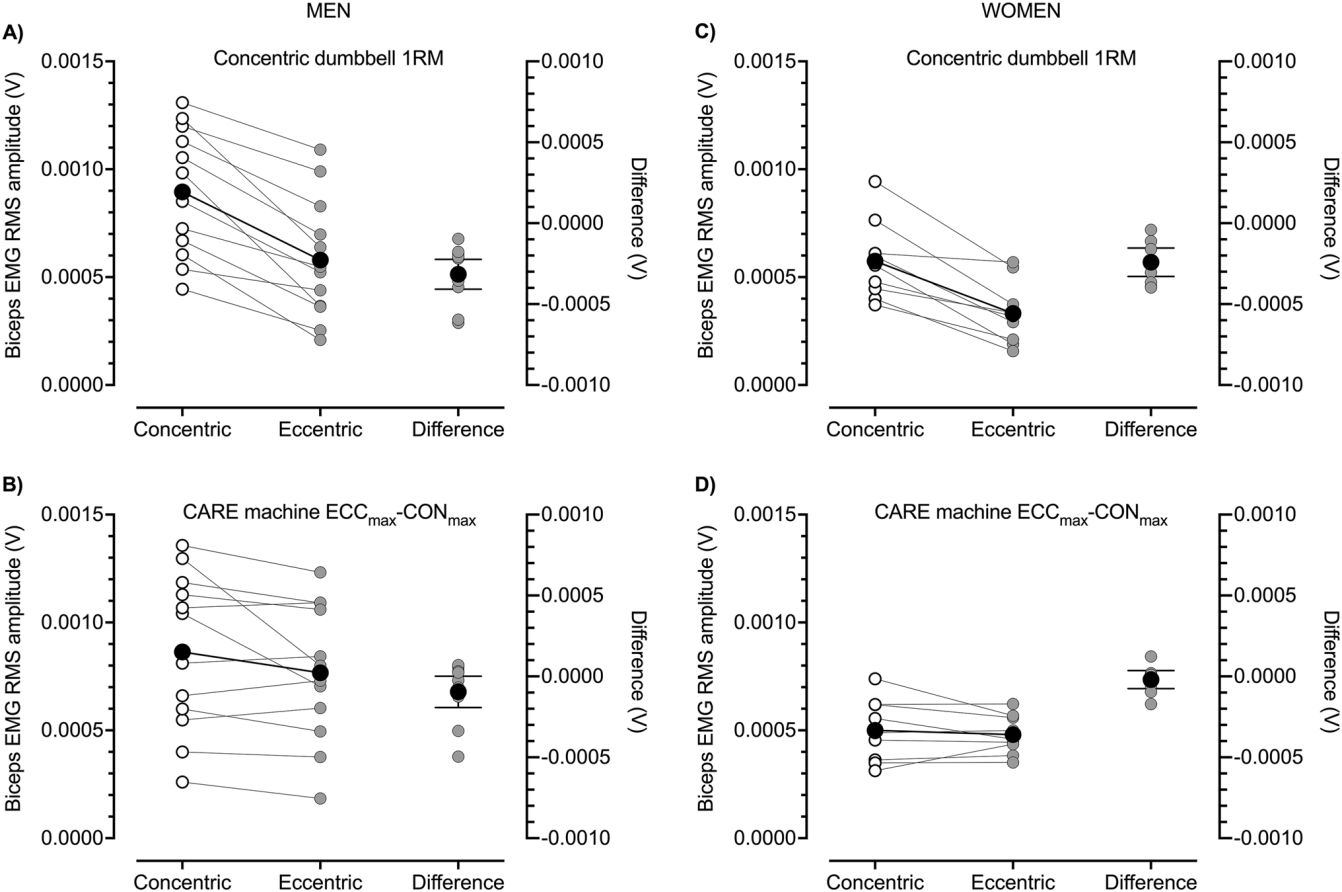
Group means (black circles) and individual participant data (white or gray circles) for root mean square (RMS) amplitudes of biceps brachii electromyographic (EMG) activity during the concentric (CON) bicep curl one repetition maximum (1RM) with a dumbbell (**A**, **C**) and during maximal eccentric (ECC), maximal CON (ECC_max_–CON_max_) repetitions on the connected adaptive resistance exercise (CARE) machine (**B**, **D**). **A** In men, biceps brachii EMG RMS amplitude was lower during the ECC than CON phase of the CON dumbbell 1RM test (mean difference [95% CIs] = − 0.000315 V [− 0.000417, − 0.000214 V]; Hedges *g* [95% CIs] = − 1.013 [− 1.589, 0.437,]). **B** In men, biceps brachii EMG RMS amplitude was similar during the ECC and CON phases of initial ECC_max_–CON_max_ repetitions on the CARE machine (mean difference = − 0.000095 V [− 0.000201, 0.000010, V; Hedges *g* = − 0.249 [− 0.550, 0.051,]). **C** In women, biceps brachii EMG RMS amplitude was lower during the ECC than CON phase of the CON dumbbell 1RM test (mean difference = − 0.000241 V [− 0.000341, − 0.000141 V]; Hedges *g* = − 1.270 [− 2.173, − 0.367]). **D** In women, biceps brachii EMG RMS amplitude was similar during the ECC and CON phases of initial ECC_max_–CON_max_ repetitions on the CARE machine (mean difference = − 0.000020 V [− 0.000083, 0.000043 V]; Hedges *g* = − 0.118 [− 0.497, 0.261])

The CARE machine provided ECC overload during ECC_max_–CON_max_ repetitions. This can be seen in the repetition-by-repetition means displayed in Fig. 1A and C and in individual data displayed in Fig. 2 (see also Table 1). These ECC_max_–CON_max_ repetitions on the CARE machine demanded high levels of neural drive. At the start of exercise, biceps brachii RMS amplitudes in the ECC phase (Fig. 3A, C), but not the CON phase (Fig. 3B, D), were greater than during the equivalent repetition phase of the dumbbell CON 1RM test. Though higher forces were generated during ECC than CON contractions on the CARE machine, biceps brachii EMG RMS amplitudes at the start of exercise were generally similar in the two phases (Fig. 4B, D). This is in contrast to the dumbbell CON 1RM test in which biceps brachii EMG RMS amplitude was lower in the ECC than CON phase (Fig. 4A, C).

**Fig. 5:**
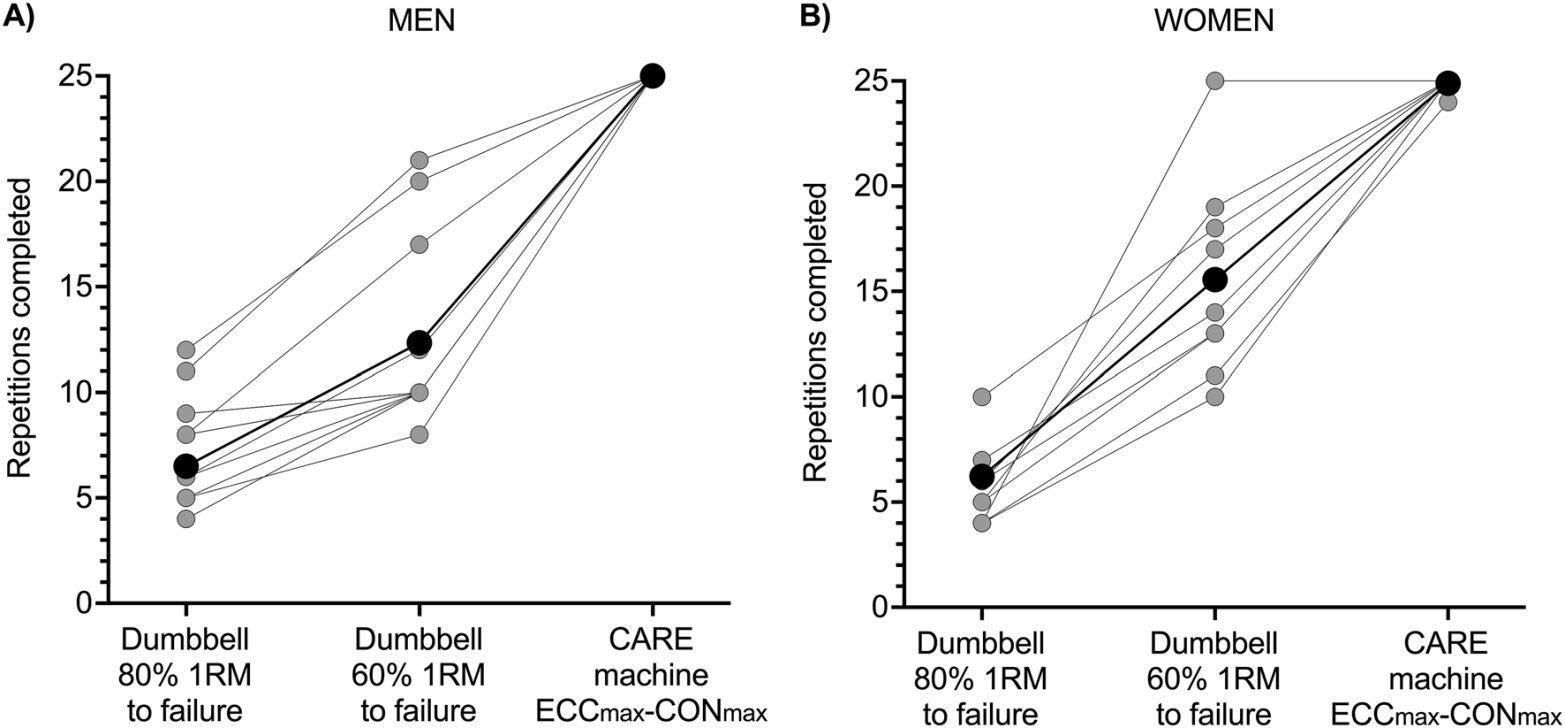
Means (black circles) and individual participant data (gray and white circles) of numbers of repetitions completed by men (**A**) and women (**B**) during one set of maximal eccentric, maximal concentric (ECC_max_–CON_max_) unilateral bicep curl exercise on the connected adaptive resistance exercise (CARE) machine and during the dumbbell repetitions-to-failure tests with loads equal to 60 and 80% of the one repetition maximum (1RM). **A** All men completed the assigned 25 ECC_max_–CON_max_ repetitions on the CARE machine without stopping. On average, men completed 12.3 and 6.5 repetitions during the repetitions-to-failure tests with dumbbells equal to 60 and 80% of 1RM, respectively. **B** All women completed the assigned 25 ECC_max_–CON_max_ repetitions on the CARE machine without stopping, except for one woman who stopped after repetition 24 due to fatigue. On average, women completed 15.6 and 6.2 repetitions during the repetitions-to-failure tests with dumbbells equal to 60 and 80% of 1RM, respectively. See Table 1 for between-sexes effect sizes

**Fig. 6:**
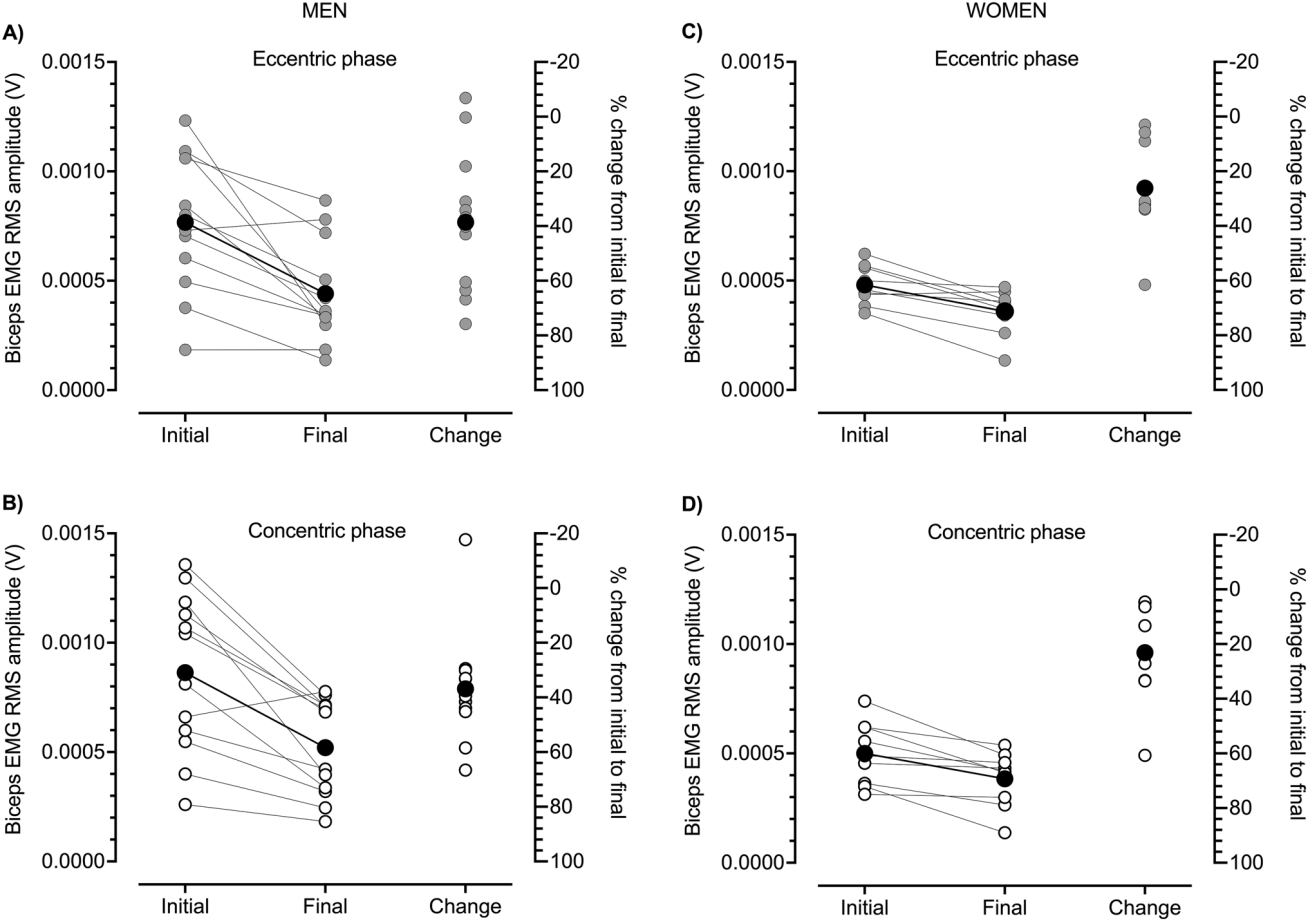
Means (black circles) and individual participant data (gray circles) for root mean square (RMS) amplitudes of biceps brachii electromyographic (EMG) activity at the start (initial) and end (final) of one set of 25 maximal eccentric, maximal concentric (ECC_max_–CON_max_) repetitions of the unilateral bicep curl on the connected adaptive resistance exercise (CARE) machine. **A** By the end of exercise, men exhibited a 39% mean reduction in ECC phase biceps brachii EMG amplitude. Not displayed is the mean raw difference [95% CI] between initial and final values which was − 0.000326 V [− 0.000505, − 0.000147 V]; Hedges *g* [95% CI] = − 1.062 [− 1.829, − 0.294]). **B** By the end exercise, men exhibited a 37% mean reduction in CON phase biceps brachii EMG amplitude. Not displayed is the mean raw difference between initial and final values which was − 0.000342 V [− 0.000503, − 0.000181 V]; Hedges *g* = − 1.192 [− 1.848, − 0.536]). **C** By the end exercise, women exhibited a 26% mean reduction in ECC phase biceps brachii EMG amplitude. Not displayed is the mean raw difference between initial and final values which was − 0.000120 V [− 0.000186, − 0.000055 V]; Hedges *g* = − 1.112 [− 1.990, − 0.234]). **D** By the end exercise, women exhibited a 23% mean reduction in CON phase biceps brachii EMG amplitude. Not displayed is the mean raw difference between initial and final values which was − 0.000116 V [− 0.000184, − 0.000048 V]; Hedges *g* = − 0.761 [− 1.387, − 0.136]). See Table 1 for between-sexes effect sizes

Due to the automatic drop setting feature of the CARE machine, nearly all participants completed the assigned 25 consecutive ECC_max_–CON_max_ repetitions without stopping, which was more repetitions than could be completed during the repetitions-to-failure tests with the 60 and 80% 1RM dumbbells (Fig. 5). This drop setting feature of the machine caused participants to experience substantial muscle fatigue. Reductions in strength were most pronounced within repetitions 1–15 after which they lessened or plateaued (Fig. 1A, C). By the end of the protocol, men lost 64 and 78% of ECC and CON phase strength, respectively (Fig. 2B, C), and women lost 44 and 63% of ECC and CON phase strength, respectively (Fig. 2E, F). Reductions in strength were accompanied by reductions in neural drive to agonist muscles, which can be seen in the repetition-by-repetition means for biceps brachii EMG RMS amplitudes displayed in Fig. 1B and D, the individual participant data displayed in Fig. 6 (see Table 1 for brachioradialis EMG data), and in raw EMG traces from one male participant displayed in Supporting Information File 2. The reduced biceps brachii EMG during the ECC_max_–CON_max_ repetitions on the CARE machine was slightly more pronounced in men (Fig. 6A, B) than women (Fig. 6C, D). Perceptions of arm strength capacity (Fig. 6A, D), biceps fatigue (Fig. 6B, E), and biceps pain (Fig. 6C, F) were also markedly changed as a result of the ECC_max_–CON_max_ repetitions with automatic drop setting. At the end of exercise, men perceived they had only 8% of their arm strength remaining, their biceps fatigue was large to extreme (7.8 of 10), and their biceps pain was small to moderate (4 of 10). Similarly, women perceived they had only 3% of their arm strength remaining at the end of exercise, their biceps fatigue was large to extreme (8.2 of 10), and their biceps pain was moderate to large (5.9 of 10).

## Discussion

We examined muscle strength, endurance, and activity, and perceptions of muscle fatigue during one set of ECC_max_–CON_max_ bicep curl exercise on a CARE machine. The main findings were that the CARE machine provided an accentuated ECC resistance and automatically reduced ECC and CON phase resistances as participants fatigued, permitting completion of 25 consecutive ECC_max_–CON_max_ repetitions without stopping. These ECC_max_–CON_max_ repetitions demanded high levels of neural drive to agonist muscles. By the end of the exercise set, both men and women exhibited significant reductions in ECC and CON phase muscle strength and EMG, and they reported heightened perceptions of fatigue in the exercised limb. However, reductions in strength and EMG were greater among the men than women, suggesting men are more susceptible to muscle fatigue from ECC_max_–CON_max_ bicep curl exercise than women.

The CARE machine safely and feasibly delivered an accentuated ECC resistance throughout exercise, confirming previous results in one individual (Nuzzo and Nosaka 2022). Thus, CARE technology has potential to facilitate the delivery of accentuated ECC resistance exercise, as coaches often prescribe ECC overload with equipment not designed for that purpose (Drury et al. 2021; Harden et al. 2020; McNeill et al. 2020). Moreover, the ECC_max_–CON_max_ repetitions on the machine demanded high levels of neural drive, which, prior to fatigue, were comparable to muscle activity achieved in the CON phase of the CON dumbbell 1RM and were greater than during the ECC phase of the CON dumbbell 1RM. This greater muscle activity during the ECC phase of CARE machine repetitions was due to the fact that the CARE machine provided a greater ECC load than the CON dumbbell 1RM. High EMG amplitudes reflect recruitment of large numbers of motor units and/or high firing rates of motor units. Thus, ECC_max_–CON_max_ repetitions on the CARE machine are associated with neurophysiological underpinnings of increased muscle strength. Nevertheless, further research is required to determine if regular use of CARE machines enhances muscle size and strength.

In the current study, the CARE machine automatically reduced resistances (i.e., drop setting) to permit completion of 25 consecutive ECC_max_–CON_max_ repetitions without stopping, confirming previous results in one individual (Nuzzo and Nosaka 2022). Thus, although each repetition phase was performed with maximal effort, the 25 repetitions were more than could be completed during the repetitions-to-failure tests with *sub*maximal dumbbells. This finding highlights the tradeoffs that occur when exercising with different equipment and loads. If an individual performs the bicep curl exercise with a near maximal dumbbell load, e.g., 80% 1RM, they will exhibit high levels of neural drive (Supporting Information 2), but they will only be able to complete ~ 5 repetitions before CON phase failure. To continue exercise, they must perform drop sets or “forced repetitions.” However, such training strategies can be inconvenient because they require the presence of a spotter or require stopping exercise to remove bar collars and plates. Individuals could, alternatively, use a moderate dumbbell, e.g., 60% 1RM, to complete more repetitions, but they will necessarily sacrifice neural drive at the beginning of the exercise set (Bigland-Ritchie 1981) (Supporting Information 2) and need to increase exercise time to perform more repetitions to recruit more motor units and/or increase motor unit firing rates (Supporting Information 3). Moreover, neither 60 nor 80% 1RM dumbbell loads provide an accentuated ECC resistance. Thus, a unique feature of the CARE machine was its ability to automate both ECC overload and drop setting. Facilitation of drop setting is important because this method of exercise increases muscle size and strength to a similar degree as other resistance exercise strategies (Ozaki et al. 2018; Varović et al. 2021). Because time is commonly cited as a barrier to exercise, and drop sets permit a high volume of work to be completed quickly, drop sets have been recommended for resistance exercise prescriptions for the general population (Iversen et al. 2021; Ozaki et al. 2020; Schoenfeld and Grgic 2018). Moreover, Ozaki et al. (2020) have argued for a specific type of drop setting called “stepwise load reduction training,” where an individual starts with a heavy load, then in a stepwise manner reduces loads for subsequent sets. Ozaki et al. (2020) proposed that this strategy might cause a broader range of physiological adaptations than traditional drop setting, which does not necessarily start with a heavy load. Results from the current study suggest that if stepwise load reduction training or other types of drop setting are effective and time-efficient strategies to increase muscle size and strength, then CARE technology can accomplish such strategies without the inconvenience of removing weight plates and without the limitation of using the same load in the ECC and CON phases.

The CARE machine’s automatic drop setting caused marked muscle fatigue. Reductions in strength occurred mostly within the first 15 repetitions, after which they tended to plateau. By the end of the protocol, CON and ECC phase strength were reduced ~ 70 and ~ 55%, respectively. These strength losses were accompanied by 20–30% reductions in biceps brachii and brachioradialis EMG, similar to reductions in EMG after fatiguing exercise with *un*coupled ECC_max_-only or CON_max_-only repetitions (Baudry et al. 2007; Komi and Rusko 1974; Pasquet et al. 2000). As expected (Bigland-Ritchie 1981), EMG profiles during the protocol on the CARE machine were different than EMG profiles during the 60 and 80% 1RM dumbbell repetitions-to-failure tests as the former was a task that started and remained maximal effort (i.e., EMG amplitude decreased over time), whereas the latter two tasks started at submaximal efforts and ended at maximal efforts (i.e., EMG amplitude increased over time) (Supporting Information 2). Reductions in strength and EMG during the protocol on the CARE machine were also accompanied by heightened perceptions of fatigue. By repetition 5, participants perceived they had already lost ~ 50% of their maximal strength capacity. By the end of the protocol, they perceived they had only ~ 5% of their strength remaining. Interestingly, though participants perceived they had almost no strength remaining in their arm at the end of the protocol, they still were generating a few kilograms of strength, particularly in the ECC phase.

The mean loss in muscle strength during the exercise protocol on the CARE machine was greater in men than women. Men lost approximately 64% of their ECC phase strength and 78% of their CON phase strength by the end of exercise. Women lost approximately 44% of their ECC phase strength and 63% of their CON phase strength by the end of exercise. Reductions in agonist EMG at the end of exercise also tended to be higher in men (35–40%) than women (25–30%). These sex differences in muscle fatigability should be interpreted with caution though due to the small sample sizes. Nevertheless, sex differences in muscle fatigability observed in the current study agree with results from studies that have included isometric exercise tasks (Hunter 2014, 2016a). One potential mechanism of greater susceptibility to muscle fatigue in men than women is greater muscle mass in men which causes greater intramuscular pressures on arteries that feed the exercising muscle and, thus, limits blood flow and oxygen supply to the muscle (Hunter 2016a). This potential mechanism is relevant for the protocol performed on the CARE machine for two reasons. One, the bicep curl exercise demands large forces from muscles of the anterior upper-limb. In these muscles, sex differences in muscle size are greater than for other muscle groups (Nuzzo 2023). Thus, the impact of muscle mass and intramuscular pressure on muscle fatigability might be exacerbated during exercise that require use of these muscles. Two, the way repetitions were performed on the CARE machine likely limited perfusion. Specifically, each repetition phase was theoretically performed at maximal tension. At the end of the ECC phase, participants immediately started the CON phase, meaning there was little opportunity for the muscles to relax between the ECC and CON phases. Also, the ECC_max_–CON_max_ repetitions on the CARE machine were performed at a slow velocity, which supports the hypothesis that men are more fatigable than women in tasks of the elbow flexors that occur at slow velocities (Hunter 2016a). Voluntary activation is another potential mechanism that might explain some of the observed sex difference in muscle fatigability (Hunter 2016a), as reductions in agonist EMG at the end of exercise tended to be greater in men than women. Other potential mechanisms for sex differences in muscle fatigability include differences in muscle fiber type, metabolism, and contractile function (Hunter 2016a; Nuzzo 2023).

**Fig. 7:**
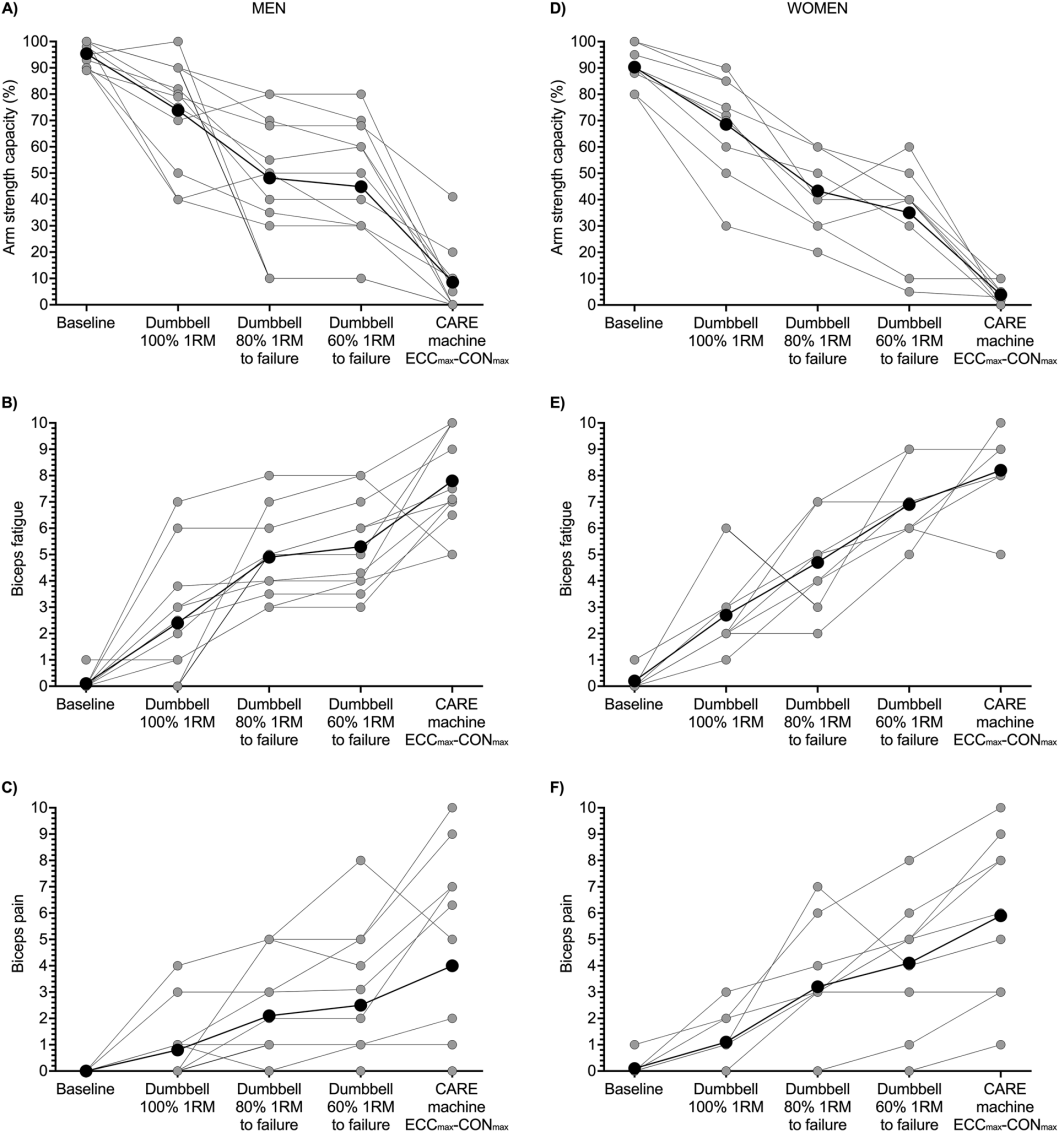
Means (black circles) and individual participant data (gray circles) for perceived arm strength capacity (**A**, **D**), biceps fatigue (**B**, **E**), and biceps pain (**C**, **F**) after one set of 25 consecutive maximal eccentric, maximal concentric (ECC_max_–CON_max_) repetitions of the unilateral bicep curl on the connected adaptive resistance exercise (CARE) machine and after repetitions-to-failure tests with dumbbells equal to 60, 80, and 100% of the one repetition maximum (1RM). **A** In men, perceived arm strength capacity was lowest after the set of 25 ECC_max_–CON_max_ repetitions on the CARE machine (8% perceived capacity remaining). Not displayed in the figure are perceived arm strength capacities for men at repetitions 5 (56.7 ± 17.3%), 10 (36.0 ± 17.4%), 15 (21.5 ± 16.0%), and 20 (12.7 ± 12.2%). **B** In men, perceived biceps fatigue was greatest after the set of 25 ECC_max_–CON_max_ repetitions on the CARE machine (i.e., large to extreme; 7.8 of 10). **C** In men, perceived biceps pain was greatest after the set of 25 ECC_max_–CON_max_ repetitions on the CARE machine (i.e., small to moderate; 4 of 10). **D** In women, perceived arm strength capacity was lowest after the set of 25 ECC_max_–CON_max_ repetitions on the CARE machine (3% perceived capacity remaining). Not displayed in the figure are perceived arm strength capacities for women at repetitions 5 (46.1 ± 12.2%), 10 (31.7 ± 6.1%), 15 (18.9 ± 6.5%), and 20 (11.3 ± 5.5%). **E** In women, perceived biceps fatigue was greatest after the set of 25 ECC_max_–CON_max_ repetitions on the CARE machine (i.e., large to extreme; 8.2 of 10). **F** In women, perceived biceps pain was greatest after the set of 25 ECC_max_–CON_max_ repetitions on the CARE machine (i.e., moderate to large; 5.9 of 10). See Table 1 for between-sexes effect sizes

The current study had limitations. The first limitation is that the male and female sample sizes were small, and more participants would have led to greater precision in the estimated sizes of effects. We chose to segregate the data by sex because roughly equal numbers of men and women volunteered to participate in the study, sex differences in muscle fatigability were possible (Hunter 2014, 2016a), and calls exist for more sex-segregated data in exercise research (Schilaty et al. 2018). Future research with larger sample sizes of men and women will help to establish more precise estimates of effects regarding muscle fatigability during ECC_max_–CON_max_ exercise on a CARE machine and during dumbbell repetitions-to-failure tests. The second limitation is that the exercise on the CARE machine was always performed after the dumbbell repetitions-to-failure tests. This is a limitation because the repetitions-to-failure tests might have impacted muscle performance and activity during subsequent exercise on the CARE machine. We chose to have participants perform the 25 consecutive ECC_max_–CON_max_ repetitions on the CARE machine last because this protocol was known from pilot testing to cause substantial fatigue and was confirmed herein (Figs. 1, 2, 7). To mitigate carryover of fatigue from one test to the next test, participants rested for 15 min between tests, similar to previous studies (Schoenfeld et al. 2014). We also assessed perceived fatigue prior to the start of each test. The average rating of perceived biceps fatigue prior to the CARE machine protocol was approximately 1.5 out of 10, suggesting participants felt little fatigue in their biceps at the time they commenced the protocol. An alternative study design might have involved completion of the CARE exercise protocol in a separate testing session. However, this would introduce issues such as requiring that the surface electrodes be removed and repositioned between testing sessions, which might impact comparisons of EMG recordings across exercise protocols. A third limitation is that the reliability of the machine’s resistances was not assessed, and this should be a focus of future investigation. Nevertheless, concurrent validity of the CARE machine was revealed in a few ways: a strong correlation (*r* = 0.964) between the dumbbell 1RM and maximal CON phase strength on the CARE machine; agonist EMG amplitude during use of the CARE machine was comparable to or greater than during the dumbbell 1RM depending on the exercise phase; resistances on the CARE machine were higher in the ECC than CON phase, which is consistent with what would be expected based on the capacity of human skeletal muscle to generate higher forces in the ECC than CON phase (Nuzzo et al. 2022); and perceptions of fatigue were heightened with actual strength loss from exercise, illustrating that the machine was continually delivering maximal or near maximal resistance to the participant. A fourth point of consideration is that drop set exercise does not necessarily increase muscle size or strength more than other resistance exercise strategies (Ozaki et al. 2018). Nevertheless, an advantage of drop set resistance exercise is that it permits a large volume of exercise to be completed in a short time, and this is potentially important because perceived lack of time is a common barrier to exercise participation (Hoare et al. 2017; Watson et al. 2006). Consequently, drop sets have been recommended as a time-efficient way to complete resistance exercise (Iversen et al. 2021; Ozaki et al. 2020).

## Practical implications

The CARE machine delivered accentuated ECC resistance exercise in a safe and feasible way. Thus, CARE technology has potential to facilitate delivery of accentuated ECC resistance exercise in research and clinical practice, particularly as inadequate equipment has been cited by coaches as a barrier to ECC resistance exercise prescriptions (Drury et al. 2021; Harden et al. 2020). The CARE machine also automated drop setting, which permitted completion of 25 ECC_max_–CON_max_ repetitions without stopping. Thus, CARE technology has potential to facilitate drop setting as a time-efficient way to complete resistance exercise (Iversen et al. 2021; Ozaki et al. 2020; Schoenfeld and Grgic 2018). Strength loss during exercise was more pronounced in men than women and suggests that men and women should not necessarily be expected to respond in the same way to acute ECC_max_–CON_max_ repetitions on a CARE machine.

## Conclusion

The novelty of the current study was the examination of muscle strength, endurance, and activity during bicep curl exercise on a new CARE machine that is commercially available for home and gym use. The main finding was that the CARE machine automated ECC overload and drop setting, such that study participants were able to complete 25 consecutive ECC_max_–CON_max_ repetitions without stopping. These maximal effort repetitions demanded high levels of neural drive, and, by the end of the exercise set, caused significant reductions in ECC and CON phase strength and EMG and heightened perceptions of muscle fatigue. Finally, men exhibited greater reductions in muscle strength and agonist EMG than women by the end of the ECC_max_–CON_max_ exercise protocol, adding to a growing literature that men appear to be more susceptible to muscle fatigue than women during some resistance exercise tasks.

## Supplementary Information

Below is the link to the electronic supplementary material.

Supplementary file1 (DOCX 122 KB)

Supplementary file2 (DOCX 4291 KB)

Supplementary file3 (DOCX 139 KB)

## Data Availability

Raw data from this study are not publicly available but some data might be available upon reasonable request made to the corresponding author.

## Abbreviations

CARE: Connected adaptive resistance exercise
CI: Confidence interval
CON: Concentric
ECC: Eccentric
EMG: Electromyography
RMS: Root mean square
SD: Standard deviation
1RM: One repetition maximum
