# Supplementary material for: Muscle strength and activity in men and women performing maximal effort biceps curl exercise on a new machine that automates eccentric overload and drop setting
Source: Eur J Appl Physiol. 2023 Mar 1;123(6):1381–96. doi: 10.1007/s00421-023-05157-9 (PMC10191922; doi:10.1007/s00421-023-05157-9)
Supplement: Supplementary file 1 — Supplementary file1 (DOCX 122 KB) [file 421_2023_5157_MOESM1_ESM.docx]

**Supporting Information 1**

Paper: Muscle strength and activity in men and women performing maximal effort bicep curl exercise on a new machine that automates eccentric overload and drop setting

Journal: *European Journal of Applied Physiology*

Authors: James L. Nuzzo, Matheus D. Pinto, Kazunori Nosaka

Email: j.nuzzo@ecu.edu.au

**Scale 1. Arm Strength Capacity Scale**

**Scale 2. Biceps Fatigue Scale**

**Scale 3. Biceps Pain Scale**
