# Supplementary material for: Muscle strength and activity in men and women performing maximal effort biceps curl exercise on a new machine that automates eccentric overload and drop setting
Source: Eur J Appl Physiol. 2023 Mar 1;123(6):1381–96. doi: 10.1007/s00421-023-05157-9 (PMC10191922; doi:10.1007/s00421-023-05157-9)
Supplement: Supplementary file 2 — Supplementary file2 (DOCX 4291 KB) [file 421_2023_5157_MOESM2_ESM.docx]

**Supporting Information 2**

Paper: Muscle strength and activity in men and women performing maximal effort bicep curl exercise on a new machine that automates eccentric overload and drop setting

Journal: *European Journal of Applied Physiology*

Authors: James L. Nuzzo, Matheus D. Pinto, Kazunori Nosaka

Email: j.nuzzo@ecu.edu.au

**
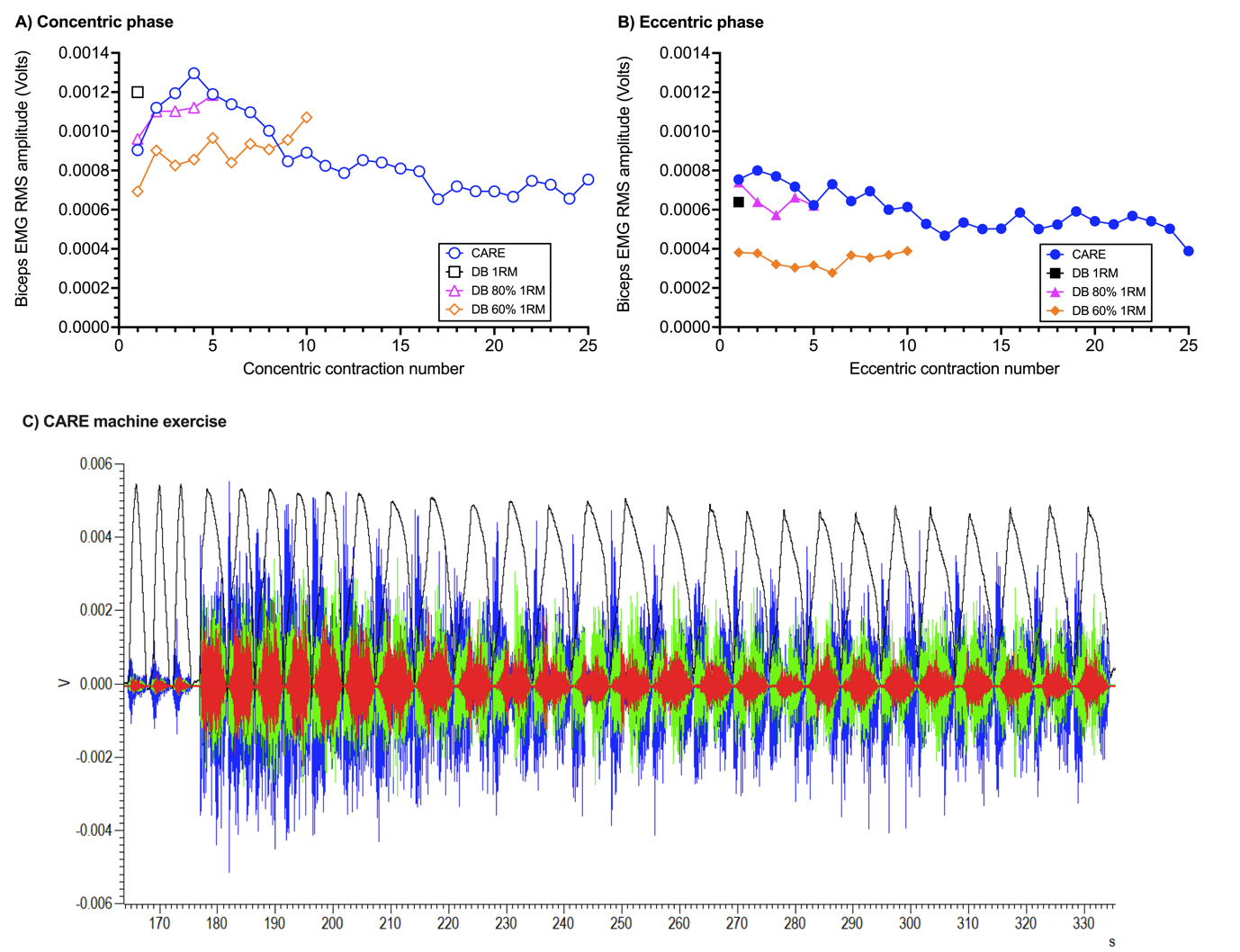
**

Raw data of electromyographic (EMG) activity from one male participant. **A:** Biceps brachii EMG root mean square (RMS) amplitude during the concentric (CON) phase of four test conditions: dumbbell one repetition maximum (DB 1RM) (black square), one set of repetitions-to-failure with an 80% DB 1RM load (pink line, triangles), one set of repetitions-to-failure with a 60% DB 1RM load (orange line, diamonds), one set of 25 consecutive eccentric maximum, concentric maximum (ECC_max_-CON_max_) repetitions on the connective adaptive resistance exercise (CARE) machine. During maximal exercise on the CARE machine, biceps brachii EMG declined progressively starting at contraction number 5 until contraction 17. During the submaximal exercise conditions (DB 60% 1RM, DB 80% 1RM), biceps brachii EMG increased progressively during the sets until repetition failure. **B:** Biceps brachii EMG RMS amplitude during the ECC phase for the same four test conditions. Biceps brachii EMG amplitude was smaller during the ECC than CON phase for all test conditions. During ECC_max_-CON_max_ repetitions on the CARE machine, biceps brachii EMG declined progressively over the 25 repetitions. **C:** Raw traces of biceps brachii (blue), brachioradialis (green), and anterior deltoid (red) EMG during the 25 consecutive ECC_max_-CON_max_ repetitions on the CARE machine. The black line represents elbow joint angle measured by the electrogoniometer. Upward deflections of the line represent CON actions and downward deflections represent ECC actions, with the top of the line representing the fully flexed elbow (i.e., end of the CON phase). This participant exhibited large EMG bursts for all three muscles early in the exercise set, with the greatest bursts occurring during repetition 4. Bursts declined noticeably in size until repetitions 9 and 10 then plateaued at a low level for the remainder of the set. When visually comparing the size of the EMG bursts in the final five repetitions to the first five repetitions, declines in EMG due to the fatiguing exercise are obvious. The three small EMG bursts on the far left of the figure represent the three calibration bicep curl repetitions that were performed with little to no external resistance prior to the start of the set of exercise on the CARE machine.
