# Supplementary material for: Muscle strength and activity in men and women performing maximal effort biceps curl exercise on a new machine that automates eccentric overload and drop setting
Source: Eur J Appl Physiol. 2023 Mar 1;123(6):1381–96. doi: 10.1007/s00421-023-05157-9 (PMC10191922; doi:10.1007/s00421-023-05157-9)
Supplement: Supplementary file 3 — Supplementary file3 (DOCX 139 KB) [file 421_2023_5157_MOESM3_ESM.docx]

**Supporting Information 3**

Paper: Muscle strength and activity in men and women performing maximal effort bicep curl exercise on a new machine that automates eccentric overload and drop setting

Journal: *European Journal of Applied Physiology*

Authors: James L. Nuzzo, Matheus D. Pinto, Kazunori Nosaka

Email: j.nuzzo@ecu.edu.au

**
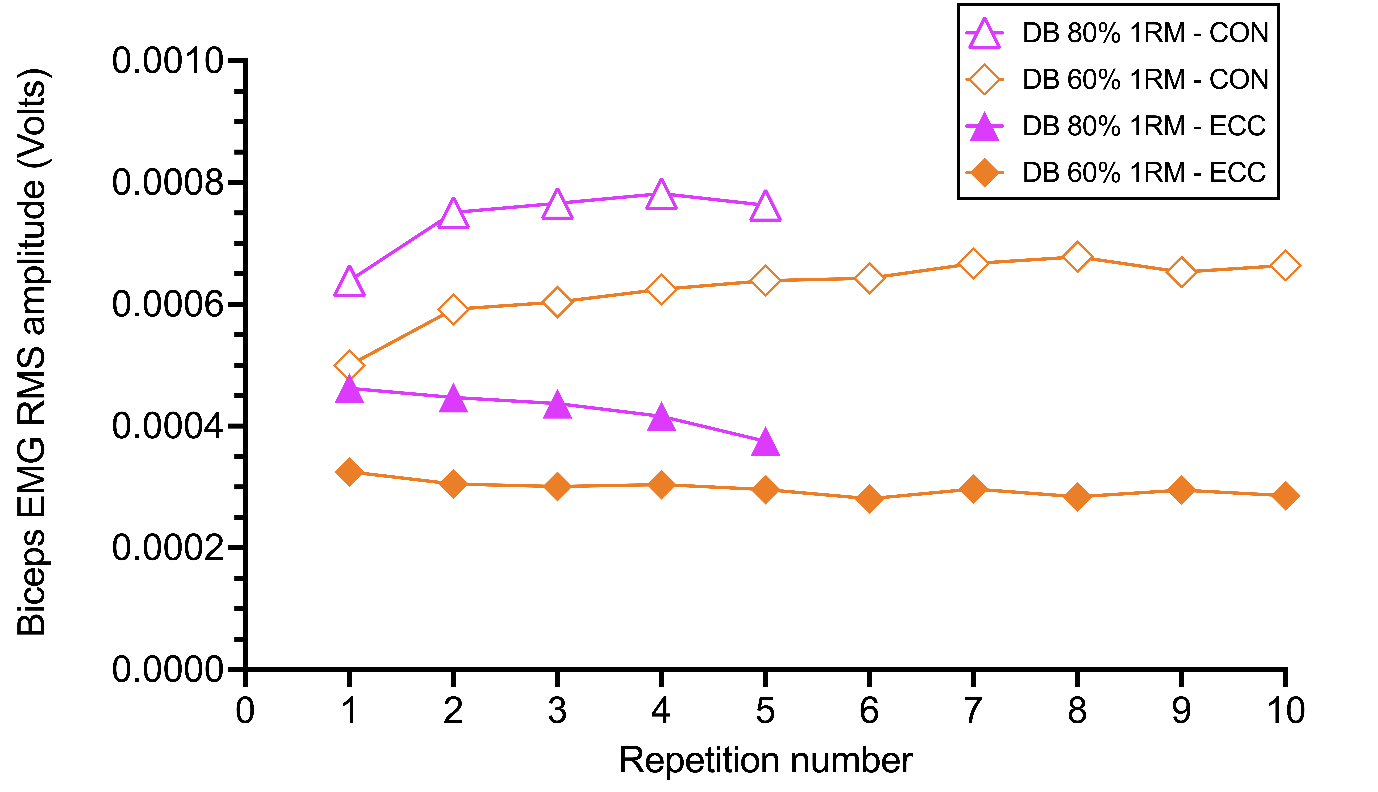
**

Means of biceps brachii electromyographic (EMG) activity during unilateral bicep curl repetitions-to-failure tests with 60% (orange line, diamonds) and 80% dumbbell one repetition maximum (DB 1RM) loads (pink line, triangles). In the repetitions-to-failure tests, participants performed different numbers of repetitions. Thus, because 20 of the 21 participants completed ≥10 repetitions with the 60% DB 1RM, EMG from the first 10 repetitions for all male and female participants were averaged and shown here. These data show during exercise with a submaximal load, concentric (CON) phase EMG amplitude starts relatively small then becomes progressively larger with each additional repetition, as participants need to recruit more motor units and/or increase their firing rates to meet task demands as fatigue accumulates. Eccentric (ECC) phase EMG amplitude was fairly stable throughout the initial 10 repetitions of the task. For the repetitions-to-failure test with the 80% DB 1RM dumbbell, 16 of 21 participants completed ≥5 repetitions. Thus, EMG data from the first 5 repetitions for all participants were averaged and are shown here. Again, CON phase EMG increased over the course of the set, whereas ECC phase EMG decreased.
